# Potentially inappropriate medicine use and predicting risk factors in hospitalized older adult patients: findings of a prospective observational study from Ethiopia

**DOI:** 10.1186/s40545-023-00663-9

**Published:** 2023-11-30

**Authors:** Behailu Terefe Tesfaye, Dula Dessalegn Bosho, Gashahun Mekonnen Dissassa, Mikiyas Gashaw Tesfaye, Mengist Awoke Yizengaw

**Affiliations:** 1 Department of Clinical Pharmacy, School of Pharmacy, Institute of Health, Jimma University, Jimma, Ethiopia; 2 Department of Internal Medicine, Jimma Medical Center, Jimma, Ethiopia; 3 Department of Laboratory, Clinical Chemistry Unit, Jimma Medical Center, Jimma, Ethiopia

**Keywords:** Aged, Inpatients, Potentially inappropriate medicine list, Ethiopia

## Abstract

**Background:**

Older patients are fragile and more susceptible to medication-related problems requiring a strict assessment of their medicine list. The present study was conducted with the intention to assess the quality use of medicines in older adult patients by detecting potentially inappropriate medicine use and its predictive risk factors.

**Methods:**

This prospective cross-sectional study involved 162 older medical patients admitted to Jimma Medical Center. A data abstraction format is employed to capture relevant information. Each patient was assessed for the presence of potentially inappropriate medicine using the 2019 American Geriatrics Associations Beers Criteria. Descriptive statistics and logistic regression analysis were conducted using STATA 15.0. A *p* value < 5% was considered a cutoff point for declaring statistical significance.

**Results:**

Over the hospital stay, 103 (63.6%) participants were on polypharmacy (5–9 concurrent medicines per patient), while 16 (9.9%) were on hyper polypharmacy (≥ 10 concurrent medicines per patient). On medicine use assessment using the Beers criteria, at least one potentially inappropriate medicine was detected in 118 (73%) participants. Overall, 191 potentially inappropriate medicines (range, 0 to 4) were identified, and 27 (14.1%) of these were associated with avoiding recommendations. Furosemide [83 (43%)], tramadol [26 (14.5%)], and spironolactone [22 (11.4%)] were the top three most frequent potentially inappropriate medicines identified. In terms of mode of prescription, 187 (96.9%) potentially inappropriate medicines were prescribed on a scheduled basis. Older adult patients with thrombocytopenia had a lower probability of taking potentially inappropriate medicine, while the odds of potentially inappropriate medicine use were 7.35 times higher in patients diagnosed with heart failure.

**Conclusions:**

Nearly three-fourths of the participants had potentially inappropriate medicine in their medicine list. Therefore, generating local evidence on the clinical, economic, and humanistic consequences may help in determining whether the Beers criteria should be taken into account when prescribing medicine to older adults. Interventions targeting older adult patients with heart failure might reduce inappropriate medicine use.

## Introduction

The global proportion of the older adult population aged 60 years and above is projected to double from 1 billion in 2020 to 2.1 billion in 2050. The estimate shows that in 2050, 80% of these older people will be living in low- and middle-income countries [1]. In Ethiopia, the proportion of these populations is also increasing over time [2]. This demographic transition is expected to have an impact on almost all aspects of society, including the healthcare system [3]. In fact, global experience shows that these age groups consume the majority of health care resources [4].

Older adult patients are usually fragile and more susceptible to drug-related problems. They are prone to multimorbidity, polypharmacy, and physiological changes that affect the kinetics and dynamics of drugs [5–7]. Evidence also suggests that these populations usually receive inappropriate medications associated with adverse consequences [8]. To mitigate this, various screening tools have been developed that assist healthcare providers in selecting medication therapy and reducing the exposure of older adult patients to potentially inappropriate medicine (PIM) [9–13]. The American Geriatric Society (AGS) Beers Criteria^®^ [9] and Screening Tool of Older People’s Potentially Inappropriate Prescriptions criteria and Screening Tool to Alert Doctors to Right Treatment (STOPP/START) [10] criteria (version 2) are the two most widely used tools to assess PIM use in older adult patients.

Beers Criteria^®^ [9] has been employed in various studies with varying reports on PIM magnitude. In a study from Europe, the prevalence of PIM ranging from 22.7% to 43.3% was reported [14]. From the Middle East, studies from Saudi Arabia [15] and the United Arab Emirates [16] revealed PIM prescriptions in 61% and 34.7% of the participants, respectively, while two studies from Kuwait recorded PIMs in 53.1% [17] and 58.4% [18] of the study participants. In Asia, studies from India reported PIM prevalence of 23.5% [19], 24.6% [20], and 61.9% [21], while PIM prescription in 34.39% of the participants was recorded in a study from China [22]. In Africa, one study from Nigeria [23] reported PIM in 31% of older adult patients, while most studies from Ethiopia reported PIM in nearly one-quarter of the participants: 27.72% from Gondar [24], 23% from Dessie [25], and 28.6% from Tigray [26]. However, in other studies from Gondar [27] and Jimma Medical Center [28], PIM was identified in 61.5% and 83.1% of older adult patients, respectively. Sex [21, 29], age [20, 21, 28, 29], educational qualification [21], polypharmacy (taking five or more medications) [14, 28, 29], hypertension [28], hospital stay for 10 or more days [19], and multiple diseases [20] are among the PIM-predicting risk factors reported in studies.

Moreover, dozens of studies have reported a significant correlation between PIM use and adverse clinical [15, 30–37] and economic [38–45] consequences. Despite ample evidence on the burden and negative consequences of PIM use in older adult patients, there are still limited studies in Ethiopia [25–28, 46, 47]. The available studies are also primarily retrospective [25, 28, 46, 47], and all of them [25–28, 46, 47] missed some pertinent covariates otherwise included in this study, such as body mass index, physical functioning and others. Furthermore, only two of the available studies were conducted in patients admitted to medical wards [25, 26]. One of the studies is from Dessie [25], which is a retrospective study prone to problems associated with the nature of the design, such as data incompleteness. The other is from Tigray [26] and considers only PIMs to be avoided in older adult patients. Hence, both studies are susceptible to understating PIM prevalence. Furthermore, both studies employed the 2012 Beers criteria in assessing PIM, which is not comprehensive in assessing PIMs compared to the latest versions [48]. Therefore, the present prospective observational study was carried out with the intention of determining the prevalence of potentially inappropriate medicine use and its associated factors in older adult patients.

## Methods

### Study design

This study is part of a prospective observational study project funded by the Jimma University Institute of Health in 2021 (JUIH2013EFY).

### Study setting

The study was carried out from 10 February 2021 to 26 December 2022 in the medical wards of Jimma Medical Center (JMC). JMC is one of the oldest public referral hospitals in Ethiopia and was established in 1930. It is located in Jimma town, 352 km southwest of Addis Ababa. JMC is the only teaching and referral hospital in the southwestern part of Ethiopia, with a bed capacity of 659. It provides services for approximately 9000 inpatient and 80,000 outpatient clients a year with a catchment population of approximately 15 million people.

### Participants’ eligibility criteria

Older adult patients aged ≥ 60 years admitted to medical wards who received at least one medication were included in the present study. The study planned to exclude older adult patients who did not consent to participate, were discharged within 24 h after admission, could not respond (patients with aphasia), and had repeated admissions during the study period. Over the actual study period, no patient refused to offer consent and was discharged within 24 h after admission.

### Study variables

The independent variables were presented in three major categories. Patient information: sex, age, residence, educational level, occupation, cigarette smoking, alcohol consumption, khat chewing, cohabitation (living arrangement), baseline body mass index (BMI), and baseline functional health status at admission. Functional health status at admission was assessed using the Katz Index of Independence in Activity of Daily Living (ADL) [49]. The tool assesses the functional health status (disability) of older individuals, ranking adequacy of performance in six functions (eating, dressing, bathing, transferring, continence and toileting). Each rank is assigned a score of 1 or 0, and the overall patient ranking is as follows: Katz score of 6 = independent (full function), 3–5 = partially dependent (moderate impairment), and 2 or less points = dependent (severe functional impairment) [49, 50]. Clinical and related information: hospitalization history in the past year, medical history, in-hospital diagnosis (disease types and number), Charlson comorbidity index (CCI) score, and length of hospital stay. The psychological condition of each patient on admission was objectively assessed using the shortened form of the Geriatric Depression Scale (GDS), which comprised 15 items [51]. Diseases were categorized according to the ICD-11 system [52], while CCI was calculated online using MDCalc [53]. Medication and related information: traditional medicine use history, past medication history, in-hospital medication, and number of in-hospital medications. The Anatomical Therapeutic Chemical (ATC) system is employed to categorize medications [54]. The outcome variables are PIM use prevalence and predicting risk factors.

### Data collection

The data collection tool was designed after reviewing the relevant literature. The tool comprised four sections: sociodemographic variables, clinical variables, medication-related variables, and outcome variables. The data collection tool was translated into the two predominant local languages (Afan Oromo and Amharic). The data collectors (two pharmacists with master's degrees in clinical pharmacy and one bachelor's degree nurse) were trained on the data collection tool and procedure. A pretest was conducted before the actual data collection. The investigators regularly supervised the data collection procedure. All eligible patients were enrolled at admission to the wards and followed until discharge. Patients were followed strictly during their hospital stay, and all relevant data were collected from the patient chart, laboratory results, patient/caregiver interviews and practitioners in charge. The weight and height of the participants were taken to calculate the body mass index (BMI) of the participants (BMI = weight in kg/(height in m)^2^. Laboratory results pertinent to judging the presence of PIM use based on the Beers criteria were extracted from the patient chart, and whenever not available in the chart, tests were requested along with other relevant tests for the patient.

### PIM assessment

In this study, current medications were assessed for potential inappropriateness. From all eligible patients, data collectors established lists of medications taken by the patient over the hospital stay. One investigator (BTT) assessed each completed questionnaire for the presence or absence of PIM using the 2019 updated American Geriatrics Associations (AGS) Beers Criteria^®^ [9]. Each assessed questionnaire was again checked for appropriateness by other investigators (MAY and DDB). The AGS Beers Criteria^®^ contains an explicit list of PIMs that are typically best avoided by older adults in most circumstances or under specific situations, such as in certain diseases or conditions. This tool is developed with the intention of improving medication selection, educating clinicians and patients, reducing ADEs, and serving as a tool for evaluating the quality of care, cost, and patterns of drug use of older adults. The criteria are comprised of five categories: medications that are potentially inappropriate in most older adults, those that should typically be avoided in older adults with certain conditions, drugs to use with caution, drug‒drug interactions, and drug dose adjustment based on kidney function. This tool has been used in previous studies from Ethiopia [24, 26, 28, 46, 47] to assess PIM use in older adult patients. In the present study, patients were considered to have been prescribed a PIM if it was prescribed before admission (admission medications) and was continued during the hospital stay or if it was newly prescribed during the hospital stay. The Beers criteria are applicable to older adult patients aged 65 years and above [9]. However, for developing countries, including Ethiopia, international organizations, such as the World Health Organization, define older adults as persons aged 60 years and above [20, 55]. Similar age cut points have also been used in various studies [20, 26]. Hence, the age cutoff point (60 years) of the present study is justified to use the Beers tool. Whenever creatinine clearance (CrCl) was needed to assess PIM according to the Beers criteria, the Cockcroft–Gault equation [56] was employed.

### Statistical methods

The sample size was determined using a single population proportion formula considering a confidence level of 95%, α = 0.05 and a critical value (Z) = 1.96. The proportions (*p* = 23%) were taken from a local-related study [25]. From the registration book review, the number of older adult patients aged 60 years and above admitted to the medical wards of JMC in 2019–2020 was considered a source population (*N* = 398). After calculating the sample size and employing a correction formula, the final calculated sample size was *n* = 162. Thus, 162 eligible older adult participants were consecutively recruited. Data completeness and accuracy were checked regularly during collection and before analysis. Each assessed PIM was double checked by the investigators. Data were entered into Epi data version 4.2.0.0 and exported to STATA 15.0 for analysis. Categorical variables were described using frequencies and percentages. Continuous variables were described using median and inter quartile (IQ). The outcome variable PIM was treated as dichotomous (1 = Yes, 0 = No) for the purpose of running a logistic regression analysis. Prior to regression analysis, a cell adequacy test was performed for each covariate. Then, running bivariable logistic regression analysis, covariates with a *p* value < 2.5 were included as candidates for the final multivariable model. A multicollinearity test was performed using the variance inflation factor (VIF). For multivariable regression analysis, fifteen covariates were identified, and all of these covariates had small (VIF < 6) and hence were retained in the model. The Hosmer‒Lemeshow goodness-of-fit test indicated a good logistic regression model fit (*p* = 0.8971). In all analyses, a *p* value < 5% was considered a cutoff value for declaring statistical significance.

## Results

### Study overview

**Fig. 1:**
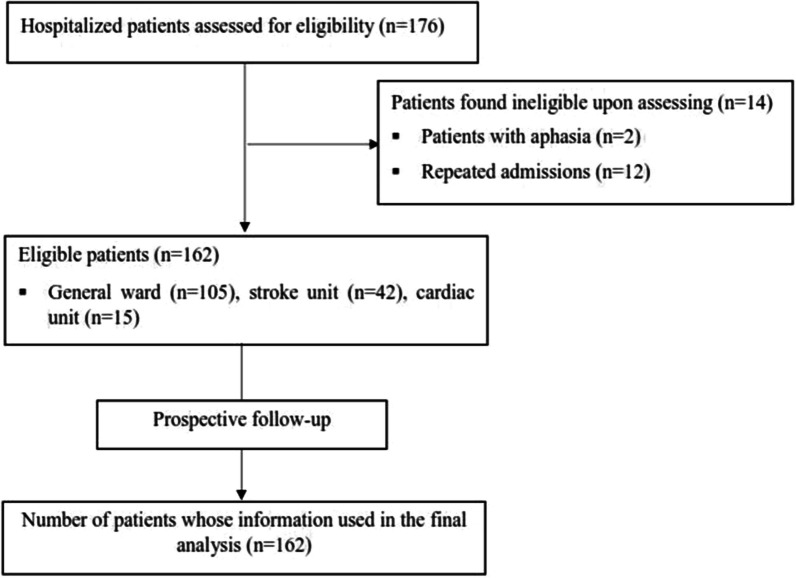
Overview of the number of patients assessed for eligibility and included in the study

During the study period, 176 hospitalized older patients were assessed for eligibility, and fourteen were ineligible. Hence, 162 participants were prospectively followed from admission until discharge, and their data were included in the final analysis (Fig. 1).

### Sociodemographic and behavioral information of the participants

The median age (IQ) of the participants was 65 (60, 70) years, and most of them were young old, 60–74 years [126 (77.8)]. The participants were predominantly males, 134 (82.7%). Above three-fourths [129 (79.6%)] of the participants were rural residents. Financially, 128 (79%) patients reported being independent for their healthcare expenditures. Only 7 (4.3%) patients reported that they lives alone. Using the Katz score for assessing ADLs, 65 (40.1) patients were found to be physically dependent (Table 1).

**Table 1:** Sociodemographic and behavioral characteristics of the study participants

Sociodemographic and behavioral variables		Frequency (%)
Age (in year)	Median (IQ)	65 (60,70)*
	60–74	126 (77.8)
	75–84	30 (18.5)
	≥ 85	6 (3.7)
Sex	Male	134 (82.7)
	Female	28 (17.3)
Residence	Urban	33 (20.4)
	Rural	129 (79.6)
Marital status	Never married	1 (0.6)
	Married	134 (82.7)
	Divorced	8 (4.9)
	Widowed	19 (11.8)
Educational level	Cannot read and write	120 (74.1)
	Nonformal education	33 (20.4)
	Primary education (1–8 grade)	6 (3.7)
	College and above	3 (1.9%)
Currently working	Yes	53 (32.7)
	No	109 (67.3)
Current occupation	Retired	20 (12.4)
	Employed	1 (0.6)
	Housewife	23 (14.2)
	Private work	52 (32.1)
	Nonemployed	66 (40.7)
Financial dependence	Dependent	34 (21)
	Independent	128 (79)
Alcohol drinking	Never	116 (71.6)
	Previously	44 (27.2)
	Current	2 (1.2)
Cigarette smoking	Never	121 (74.7)
	Ex-smoker	39 (24.1)
	Current	2 (1.2)
Khat chewing	Never	45 (27.8)
	Previously	105 (64.8)
	Current	12 (7.4)
Traditional medicine use history	Yes	21 (13)
	No	141 (87)
Cohabitation	Live with spouse and children	85 (52.5)
	Live with spouse	41 (25.3)
	Live with children	29 (17.9)
	Live alone	7 (4.3)
Activities of daily living	Median (IQ) Katz Score	3.5 (0,6)*
	Dependent	65 (40.1)
	Partially dependent	51 (31.5)
	Fully independent	46 (28.4)
BMI, kg/m^2^	Median (IQ)	19.5 (17.8, 20.7)*
	Underweight (less than 18.5)	46 (28.4)
	Normal (18.5 to < 25)	107 (66.1)
	Overweight (25.0 to < 30)	9 (5.6)

*Median (IQ)

*BMI* body mass index, *IQ* interquartile

### Clinical and related information of the participants

Of the total, 105 (64.8%) participants had a past medical history. Diseases of the circulatory system were the most frequent disease categories diagnosed in the study period [112 (69.1%)]. Approximately one-third [53 (32.8%)] of the participants experienced a minimum of one hospitalization history within the past 1 year before the study period (Table 2).

**Table 2:** Clinical and related characteristics of the study participants

Clinical and related information	Frequency (%)
Patients with previous medical history	105 (64.8)
Hospitalization in the previous 1-year before the study period	
None	109 (67.3)
Ones	49 (30.3)
Twice and above	4 (2.5)
Psychological condition on admission (GDS score)	
No psychological problems (0 to 4)	34 (21%)
Mild dementia/depression (5 to 9	96 59.3%)
Severe dementia/depression (10 to 15)	32 (19.8%)
Currently diagnosed diseases according to ICD-11 classification	
Certain infectious or parasitic diseases	20 (12.4)
Neoplasms	3 (1.9)
Diseases of the immune system	5 (3.1)
Endocrine, nutritional or metabolic diseases	35 (21.6)
Mental, behavioral or neurodevelopmental disorders	2 (1.23)
Diseases of the nervous system	21 (13)
Diseases of the circulatory system	112 (69.1)
Diseases of the respiratory system	70 (43.2)
Diseases of the digestive system	12 (7.4)
Diseases of the skin	1 (0.6)
Diseases of the blood or blood-forming organs	33 (20.4)
Diseases of the genitourinary system	37 (22.8)
Symptoms, signs or clinical findings, not elsewhere classified	13 (8)
Number of diseases diagnosed	
Median (IQ)	3 (3, 4)
1	8 (4.9)
2	30 (18.5)
3	46 (28.4)
4	39 (24.1)
5 and above	39 (24.1)
CCI score	
Median (IQ)	4 (3, 5)
Mild	12 (7.4)
Moderate	93 (57.4)
Severe	57 (35.2)
Length of hospital stay, days	
Median (IQ)	10 (6, 14)
Short stays (0–5 days)	24 (14.8)
Medium stays (6–10 days)	67 (41.4)
Long stay (≥ 10 days)	71 (43.8)

*CCI* Charlson comorbidity index, *ICD-11* International Classification of Diseases 11th Revision, *IQ* interquartile

**Fig. 2:**
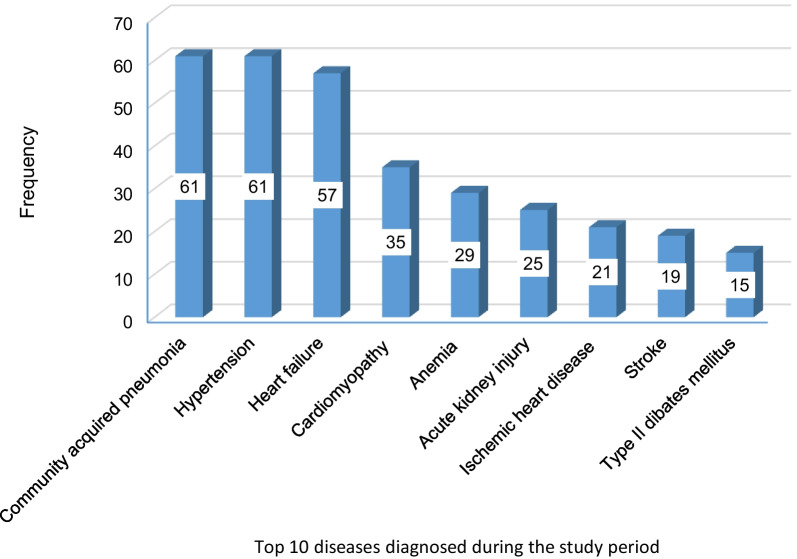
Top 10 diseases diagnosed in older adult patients over hospital stay

Community-acquired pneumonia (*n* = 61), hypertension (*n* = 61), and heart failure (*n* = 57) were the three most frequent diagnoses over the hospital stay (Fig. 2).

### Medication and related information of the participants

Medication use in the past 3 months before the study period was reported in nearly half [75 (46.3%)] of the participants. Regarding past medication history, cardiovascular system medication use was reported in 38 (23.5%) patients. Over the hospital stay, polypharmacy (≥ 5 medications) was noted in 109 (63.6%) patients (Table 3).

**Table 3:** Medication and related information of the participants

ATC code	Medications category according to ATC	In-hospital medications, *n* (%)
A	Alimentary tract and metabolism	90 (55.6)
B	Blood and blood forming organs	98 (60.5)
C	Cardiovascular system	120 (74.1)
H	Systemic hormonal preparations	32 (19.8)
J	Anti-infective for systemic use	110 (67.9)
M	Musculo-skeletal system	2 (1.2)
N	Nervous system	40 (24.7)
P	Antiparasitic products, insecticides and repellents	1 (0.6)
R	Respiratory system	29 (17.9)
V	Various agents	4 (2.5)
In hospital medications		
Median (IQ) number		6 (4, 7)
1–4		43 (26.5)
5–9		103 (63.6)
≥ 10		16 (9.9)
Total number		989

*ATC* anatomical therapeutic chemical, *IQ* interquartile

### Potentially inappropriate medicine prescription

In the assessment of in-hospital medications using the 2019 Beers criteria, at least one PIM use was detected in 118 (73%) participants. The total number of PIMs was 191, of which the Beers criteria recommend avoidance of 27 (14.1%) (Table 4).

**Table 4:** Prevalence of potentially inappropriate medicine and related information

PIM prescription over hospital stay	
Patients with PIM, *n* (%)	118 (73)
Total number of PIMs	191
Median (IQ) number of PIM per patient	1 (0, 2)
Minimum, Maximum PIM per patient	0, 4
Mode of PIM prescription, *n* (%)	
Scheduled	185 (96.9)
As needed	6 (3.1)
Overall beers recommendations, *n* (%)	
Avoid	27 (14.1)
Use with caution	133 (69.6)
Reduce dose	31 (16.2)

*IQ* interquartile, *PIM* potentially inappropriate medication

The three most frequent PIMs identified were furosemide [83 (43%)], tramadol [26 (14.5%)] and spironolactone [22 (11.4%)]. (Table 5).

**Table 5:** Specific Beers PIM with recommendations and reasons

Specific PIMs	Frequency (%)	Beers recommendation	Reason (s)
Amitriptyline	2 (1.0)	Avoid	Highly anticholinergic, sedating, and cause orthostatic hypotension
Aspirin	1 (0.5)	Use with caution in patients ≥ 70 years	Aspirin for primary prevention of cardiovascular disease
Warfarin and Aspirin	3 (1.5)	Avoid when possible; if used together, monitor INR closely	Increased risk of bleeding
Cimetidine	14 (7.2)	Reduce dose if CrCl is < 50	Mental status changes
Warfarin and Ciprofloxacin	2 (1.0)	Avoid when possible; if used together, monitor INR closely	Increased risk of bleeding
Dexamethasone and NSAID	1 (0.5)	Avoid; if not possible, provide gastrointestinal protection	Increased risk of peptic ulcer disease or gastrointestinal bleeding
Digoxin	4 (2.1)	Avoid this rate control agent as first line therapy for atrial fibrillation	Should not be used as a first-line agent in atrial fibrillation, because there are safer and more effective alternatives for rate control
Furosemide	83 (43)	Use with caution	May exacerbate or cause SIADH or hyponatremia; monitor sodium level closely when starting or changing dosages in older adults
Hydrochlorothiazide	4 (2.1)	Use with caution	May exacerbate or cause SIADH or hyponatremia; monitor sodium level closely when starting or changing dosages in older adults
Metoclopramide	9 (4.7)	Avoid, unless for gastroparesis with duration of use not to exceed 12 weeks except in rare cases	Can cause extrapyramidal effects, including tardive dyskinesia; risk may be greater in frail older adults
Ranitidine	17 (8.8)	Reduce dose if CrCl is < 50	Mental status changes
Sliding-scale regular Insulin alone	2 (1.0)	Avoid	Insulin regimens that include only short- or rapid acting insulin increases the risk of hypoglycemia without improvement in hyperglycemia management regardless of care setting
Risperidone	1 (0.5)	Avoid	Avoid in older adults with or at high risk of delirium because of potential of inducing or worsening delirium
Spironolactone	22 (11.4)	Avoid in patients with CrCl < 30 = 2	Increased potassium
		Use with caution = 20	May exacerbate or cause SIADH or hyponatremia; monitor sodium level closely when starting or changing dosages in older adults
Tramadol	26 (14.5)	Avoid if CrCl < 30 = 1	CNS adverse effect
		Use with caution = 25	May exacerbate or cause SIADH or hyponatremia; monitor sodium level closely when starting or changing dosages in older adults

*CNS* central nervous system, *CrCl* creatinine clearance, *IQ* interquartile, *PIM* potentially inappropriate medication, *SIADH* syndrome of inappropriate secretion of antidiuretic hormone

### Predictors of inappropriate medication use

In the final multivariable model, thrombocytopenia and heart failure diagnosis were significantly associated with PIM prescription. Older patients with thrombocytopenia were at a lower risk of receiving PIMs, while the odds of taking PIM were 7.35 times higher in patients diagnosed with heart failure (Table 6).

**Table 6:** Bivariable and multivariable logistic regression analyses

Variables	PIM (Yes)	PIM (No)	Bivariable analysis		Multivariable analysis	
			COR (95%CI)	*p* value	AOR (95%CI)	*p* value
Sex						
Male	93 (78.81)	41 (93.18)	1		1	
Female	25 (21.19)	3 (6.82)	3.67 (1.05, 12.86)	**0.042**	2.69 (0.49, 14.92)	0.255
Khat chewing				0.155		
Never	35 (29.66)	10 (22.73)	1		1	
Former	77 (65.25)	28 (63.64)	0.79 (0.34, 1.79)	0.567	0.60 (0.19, 1.9)	0.388
Current	6 (5.08)	6 (13.64)	0.29 (0.07, 1.08)	0.065	0.25 (0.04, 1.74)	0.162
Activities of daily living				0.105		
Dependent	42 (35.59)	23 (52.27)	0.38 (0.15, 0.96)	0.041	0.59 (0.16, 2.23)	0.441
Partially dependent	38 (32.20)	13 (29.55)	0.62 (0.23, 1.65)	0.336	0.51 (0.14, 1.86)	0.305
Fully independent	38 (32.20)	8 (18.18)	1		1	
Acute kidney injury						
Yes	21 (17.80)	4 (9.09)	2.16 (0.69, 6.71)	0.181	1.59 (0.38, 6.66)	0.528
No	97 (82.20)	40 (90.91)	1		1	
Asthma						
Yes	5 (4.24)	4 (9.09)	0.44 (0.11, 1.73)	0.241	0.12 (0.01, 0.17)	0.068
No	113 (95.76)	40 (90.91)	1		1	
Community acquired pneumonia						
Yes	50 (42.37)	11 (25)	2.21 (1.02, 4.78)	**0.045**	0.77 (0.26, 2.23)	0.625
No	68 (57.63)	33 (75)	1		1	
Heart failure						
Yes	55 (46.61)	2 (4.55)	18.33 (4.24, 79.25)	**0.000**	7.35 (1.25, 43.2)	**0.027**
No	63 (53.39)	42 (95.45)	1		1	
Hemiplegia						
Yes	6 (5.08)	5 (11.36)	0.42 (0.12, 1.45)	0.168	3.66 (0.39, 34.43)	0.256
No	112 (94.92)	39 (88.64)	1		1	
Systemic hypertension						
Yes	42 (35.59)	19 (43.18)	0.73 (0.36, 1.47)	0.376	0.64 (0.18, 2.27)	0.490
No	76 (64.41)	25 (56.82)	1		1	
Stroke						
Yes	7 (5.93)	12 (27.27)	0.17 (0.06, 0.46)	**0.001**	0.17 (0.02, 1.57)	0.119
No	111 (94.07)	32 (72.73)	1		1	
Pulmonary hypertension						
Yes	10 (8.47)	1 (2.27)	3.98 (0.49, 32.05)	0.194	1.07 (0.09, 13.43)	0.956
No	108 (91.53)	43 (97.73)	1		1	
Thrombocytopenia						
Yes	4 (3.39)	6 (13.64)	0.22 (0.06, 0.83)	**0.025**	0.17 (0.03, 1.88)	**0.035**
No	114 (96.61)	38 (86.36)	1		1	
Cardiomyopathy						
Yes	33 (27.97)	2 (4.55)	8.15 (1.87, 35.61)	**0.005**	2.55 (0.34, 18.9)	0.360
No	85 (72.03)	42 (95.45)	1		1	
Number of diseases, median (IQ)	4 (3, 5)	3 (2, 4)	1.52 (1.16, 1.99)	**0.002**	1.28 (0.89, 1.84)	0.188
1	5 (4.2)	3 (6.8)	1			
2	15 (12.7)	15 (34.1)	0.6 (0.12, 2.97)	0.532	0.92 (0.13, 6.65)	0.937
3	34 (28.8)	12 (27.3)	1.7 (0.35, 8.21)	0.509	2.58 (0.35, 18.97)	0.351
4	29 (24.6)	10 (22.7)	1.74 (0.35, 8.63)	0.498	1.47 (0.2, 10.8)	0.705
≥ 5	35 (29.7)	4 (9.1)	5.25 (0.9, 30.7)	0.066	4.99 (0.49, 51.27)	0.176
Number of in hospital medications						
1–4	22 (18.6)	21 (47.7)	1	**0.000**		
5–9	81 (68.6)	22 (50)	3.51 (1.64, 7.52)	**0.001**	2.25 (0.84, 6.01)	0.107
≥ 10	15 (12.7)	1 (2.3)	14.32 (1.73, 118.18)	**0.013**	10.75 (0.99, 116.2)	0.051

*AOR* adjusted odd ratio, *COR* crude odd ratio, *PIM* potentially inappropriate medication

## Discussion

In this prospective cross-sectional study, patients were followed from admission to different units of medical wards until discharge. At least one PIM use was detected in 118 (73%) participants. This shows that the quality of medicine use is poor in approximately three-fourths of admitted older adult patients. In a similar study from India, inappropriate medication use was detected in 61.9% of the participants [21]. By far lower PIM prevalence, 23.5% [19] and 24.6% [20] were recorded in other studies from India. Both of these studies employed the 2003 version of the Beers criteria. Furthermore, in a study by Nagendra [20], all the clinicians of general medicine wards were informed on the use of beers criteria to identify PIMs. This potentially alert prescribers/clinicians to reduce prescribing inappropriate medications in the study period. Hence, among other potential confounders, the nonblinded approach [20] and the difference in the version of Beers criteria employed might have resulted in a lower PIM prevalence report compared to our present finding. Similarly, studies from the United Arab Emirates [16] and China [22] reported PIM prescriptions in 34.7% and 34.39% of the participants, respectively, which is lower than our findings. This discrepancy could partly be explained by the variation in study design. The study from the United Arab Emirates [16] included elderly patients who were prescribed 5 or more medications and were discharged [16], whereas in our study, elderly patients who received at least one medication were included, and the medications assessed for PIM were those taken over the inpatient stay. On the other hand, the study from China [22] was conducted in outpatient settings.

In Ethiopia, two previous similar studies reported PIM prevalence in less than one-third of the participants: 23% from Dessie [25] and 28.6% from Tigray [26]. As can clearly be seen in the method section, the present study assessed PIMs using the 2019 Beers criteria and considered all types of PIMs (avoid, use with caution, and reduce dose) in the assessment and report. However, an earlier study from Tigray [26] assessed PIMs using the 2012 Beers criteria and considered only PIMs to be avoided in older adult patients. This underestimated the overall PIM prevalence rate in their study. In fact, in the present study, PIMs to be avoided were identified in 27 (14.1%) patients, which is half as low as a study report from Tigray [26]. On the other hand, a study from Dessie [25] is a retrospective study. The intrinsic nature of a retrospective study, such as data incompleteness, might have led to understated PIM magnitude.

In the present study, thrombocytopenia and heart failure diagnoses were independently associated with PIM prescription. Accordingly, older patients with thrombocytopenia were less likely to receive PIMs than those who did not, whereas those patients diagnosed with heart failure had a 7.75-fold increased risk of taking PIMs than their counterparts. This could be due to the potential difference in the number and type of medications prescribed in patients with various diagnoses.

In this study, although the odds of receiving PIM were found to be high in patients on polypharmacy and hyper polypharmacy, both failed to achieve statistical significance. In fact, concurrent use of multiple medications could increase the risk of drug‒drug and drug–disease interactions as well as complicate quality of care, resulting in a higher probability for PIM prescriptions [28]. This relationship has been confirmed in multiple studies from various geographical regions [14, 17, 19, 20, 25, 26, 29]. In studies from Europe [14], Kuwait [17], and Ethiopia [25, 26], polypharmacy, defined as concurrently taking 5 or more medicines, is reported as a risk factor for a significant increase in PIM prescription. Studies from India reported a significant increase in the risk of PIM use in patients concurrently taking 9 or more [19] and 10–14 [20] medicines. However, polypharmacy, as reported in the aforementioned studies [14, 17, 25, 26], was not significantly correlated with PIM use in the present study.

Other studies have reported an increased risk of PIM use in female patients [21, 29], with an increase in age [29], in patients with educational qualifications of 11th–12th class [21], with a hospital stay ≥ 10 days [19], and in patients with multiple diseases (≥ 4) [20]. Likewise, in the present study, female patients and patients with a higher number of diseases were more likely to receive PIMs. Both of these factors were significantly associated with PIM use in the binary regression; however, the association was lost after adjusting for other candidate variables in the final model. Otherwise, age and educational qualification were not correlated with PIM use, even in the binary regression analysis in our study. This could be attributed to the small sample size employed in the present study.

From the findings of the present study, the investigators suggest that healthcare providers be vigilant in prescribing medications to older patients. Healthcare providers are also recommended to consider the PIM assessment tool as a means to ensure the quality use of medicine in this age group. On the other hand, policy makers are recommended to pay attention to the quality use of medicine in elderly patients while drafting healthcare guidelines and directives. Developing and installing key performance indicators on medication use quality in healthcare institutions might also be helpful.

To the best of the authors’ knowledge, this study is the first to comprehensively identify PIMs and assess potential explanatory variables in older adult patients admitted to all medical units in a healthcare setting in Ethiopia. Its prospective nature, use of the latest version PIM assessing tools by the time, and consideration of important but usually missed covariates are among the merits of the present study. However, the small sample size employed and consideration of only a single institution could affect the generalizability and power of the study.

## Conclusion

Potentially inappropriate medicine use was detected in nearly three-fourths of the older patients. Therefore, assessing the clinical and economic consequences of PIM use in the local context; considering, adapting and employing Beers criteria in medicine prescribing practice for older adult patients; and tailoring interventions targeting patients with heart failure might help reduce PIM use in older adult patients.

## Data Availability

The data sets used and/or analysed during the current study are available from the corresponding author upon reasonable request.

## Abbreviations

ADL: Activity of daily living
AOR: Adjusted odd ratio
ATC: Anatomical therapeutic chemical
BMI: Body mass index
CCI: Charlson comorbidity index
CNS: Central nervous system
COR: Crude odd ratio
CrCl: Creatinine clearance
GDS: Geriatric depression scale
ICD-11: International Classification of Diseases 11th Revision
IQ: Inter quartile
JMC: Jimma Medical Center
PIM: Potentially inappropriate medicine
STOPP/START: Potentially inappropriate prescriptions criteria and screening tool to alert doctors to right treatment
VIF: Variance inflation factor
